# Decreased human respiratory syncytial virus activity during the COVID-19 pandemic in Japan: an ecological time-series analysis

**DOI:** 10.1186/s12879-021-06461-5

**Published:** 2021-08-03

**Authors:** Keita Wagatsuma, Iain S. Koolhof, Yugo Shobugawa, Reiko Saito

**Affiliations:** 1 Division of International Health (Public Health), Graduate School of Medical and Dental Sciences, Niigata University, 1-757 Asahimachi dori, Chuo-ku, Niigata City, 951-8510 Japan; 2 College of Health and Medicine, School of Medicine, University of Tasmania, Hobart, Australia; 3 Department of Active Ageing (donated by Tokamachi city, Niigata, Japan), Graduate School of Medical and Dental Sciences, Niigata University, Niigata, Japan

**Keywords:** COVID-19, SARS-CoV-2, HRSV, NPIs, Epidemics

## Abstract

**Background:**

Non-pharmaceutical interventions (NPIs), such as sanitary measures and travel restrictions, aimed at controlling the severe acute respiratory syndrome coronavirus 2 (SARS-CoV-2), may affect the transmission dynamics of human respiratory syncytial virus (HRSV). We aimed to quantify the contribution of the sales of hand hygiene products and the number of international and domestic airline passenger arrivals on HRSV epidemic in Japan.

**Methods:**

The monthly number of HRSV cases per sentinel site (HRSV activity) in 2020 was compared with the average of the corresponding period in the previous 6 years (from January 2014 to December 2020) using a monthly paired *t*-test. A generalized linear gamma regression model was used to regress the time-series of the monthly HRSV activity against NPI indicators, including sale of hand hygiene products and the number of domestic and international airline passengers, while controlling for meteorological conditions (monthly average temperature and relative humidity) and seasonal variations between years (2014–2020).

**Results:**

The average number of monthly HRSV case notifications in 2020 decreased by approximately 85% (*p* < 0.001) compared to those in the preceding 6 years (2014–2019). For every average ¥1 billion (approximately £680,000/$9,000,000) spent on hand hygiene products during the current month and 1 month before there was a 0.29% (*p* = 0.003) decrease in HRSV infections. An increase of average 1000 domestic and international airline passenger arrivals during the previous 1–2 months was associated with a 3.8 × 10^− 4^% (*p* < 0.001) and 1.2 × 10^− 3^% (*p* < 0.001) increase in the monthly number of HRSV infections, respectively.

**Conclusions:**

This study suggests that there is an association between the decrease in the monthly number of HRSV cases and improved hygiene and sanitary measures and travel restrictions for COVID-19 in Japan, indicating that these public health interventions can contribute to the suppression of HRSV activity. These findings may help in public health policy and decision making.

**Supplementary Information:**

The online version contains supplementary material available at 10.1186/s12879-021-06461-5.

## Background

The human respiratory syncytial virus (HRSV) is an infection of the respiratory tract which causes clinically severe pneumonia in young children and bronchitis in infants [1]. Globally, acute lower respiratory tract infections (ALRIs) caused by HRSV lead to the deaths of approximately 70,000 children under the age of 5 years annually, and with approximately 3.4 million people requiring hospitalization worldwide [2, 3]. Recently, the global burden of disease caused by HRSV has become more apparent, not only in infants and young children, but also in the elderly (≥65 years); furthermore, no effective vaccine has yet been developed, leaving a significant clinical impact [4, 5].

The coronavirus disease 2019 (COVID-19), caused by the severe acute respiratory syndrome coronavirus 2 (SARS-CoV-2), is currently an unprecedented global pandemic [6, 7]. As of late February 2021, more than 108.2 million confirmed cases of COVID-19 have been reported worldwide, with over 2.3 million deaths [8]. Japan experienced three COVID-19 epidemic peaks by October 2020 [9], an increase in cases was expected, and an effective approach to slow the spread of the virus was sought-after.

The adoption of non-pharmaceutical interventions (NPIs), including sanitary measures and travel restrictions, is crucial for reducing the transmission of respiratory infections such as COVID-19, especially before effective vaccines become widely available [10–13]. The World Health Organization (WHO) has recommended NPIs such as hand hygiene, social distancing measures, and use of face masks to reduce COVID-19 transmission [14]. These hygiene measures provide a good opportunity to promote universal disease control and prevention measures in local communities [15].

Interventions aimed at reducing the impact of the COVID-19 pandemic may potentially affect the epidemic dynamics of other respiratory infections [16]. In Japan, contagious viral respiratory diseases such as seasonal influenza and HRSV commonly cause epidemics. For seasonal influenza, a downward trend was reported in Brazil, Singapore, Taiwan, and Japan during the COVID-19 pandemic, indicating that seasonal influenza activity was less in 2020 than in previous years [16–20]. Even in the southern hemisphere, such as Australia, no historical summer outbreaks were reported, and seasonal influenza activity was low [21]. Evidence reporting the potential effect of public health interventions during the COVID-19 pandemic on the reduction of HRSV activity is scarce and unclear.

To understand the potential effect of NPIs for COVID-19 prevention on HRSV activity in Japan, epidemiological analysis of the transmission dynamics of HRSV in relation to NPI indicators is needed. The potential effects of increased hygiene practices and decreased human movement brought about by the COVID-19 pandemic may extend to the reduction of other viral infections at the local level. The main aim of the present study is to determine the effects of NPIs on HRSV activity. Here, we compared HRSV activity in Japan during 2020 to that in the preceding 6 epidemiologic years (2014–2019) to identify the current characteristics of HRSV activity. We further investigated associations between HRSV activity and the indicators for NPIs, such as the sale of hand hygiene products and the number of international and domestic airline passenger arrivals over the study period (2014–2020). This study provides basic epidemiological information on whether adherence to NPIs for COVID-19 prevention contributed to the reduction of HRSV transmissibility in Japan in 2020.

## Methods

### National HRSV surveillance data

The HRSV epidemiological data used in this study was obtained from the Infectious Disease Weekly Report (IDWR), which was sourced from the National Epidemiological Surveillance of Infectious Diseases (NESID) data published by the National Institute of Infectious Diseases, Japan (NIID) under the Ministry of Health, Labor and Welfare, Japan (MHLW) [22]. The MHLW manages approximately 3000 pediatric sentinel sites (i.e., hospitals and clinics) in Japan, which report the number of patients diagnosed with an HRSV infection on a weekly basis to the prefecture or municipal public health sectors in Japan [23, 24]. A confirmed case of HRSV infection is defined by a positive result in a rapid diagnostic test (RDT) kit licensed in Japan, or a laboratory confirmation such as virus isolation or antibody titer increase in paired sera according to the MHLW guidelines [25]. The number of sentinels assigned to each public health service area is determined based on population size: a public health centre with < 30,000 individuals has one sentinel, a centre with 30,000–75,000 individuals has two sentinels, and one with > 75,000 individuals has ≥3 sentinels, as determined by the following formula: 3 + (population–75,000)/50,000 [26]. These sentinel sites forward clinical data to approximately 60 prefectural or municipal public health sectors, and these data are electronically reported to the NIID; the number of HRSV cases are released weekly through its online website. In this present study, we extracted the total number of HRSV cases per sentinel site (HRSV activity) at a national level in Japan reported in weeks 1–52, in 2014–2020 from the NESID. The monthly HRSV activity at national level in Japan were compiled based on these weekly HRSV data.

### NPI indicators

#### Retail sales of hand hygiene products

To evaluate the level of hand hygiene behavior (i.e., potential effect of sanitary measures), we used data regarding the monthly retail sales of hand hygiene products (hand soap and ethyl alcohol) per ¥1 billion (approximately £6800,000/$9,000,000) (unit: yen) as an explanatory variable in the models presented here. These were extracted from the statistics of production in the chemical industries under the Ministry of Economy, Trade and Industry, Japan (METI) for the 7 epidemiologic years (2014–2020); these values were summed and used as an indicator of retail sales of hand hygiene products [27].

#### International and domestic airline passenger arrivals data

To evaluate the potential effect of travel restrictions, we used the data regarding the monthly number of international and domestic airline passenger arrivals per 1000 population (unit: person) in Japan for the 7 epidemiologic years (2014–2020) as an explanatory variable in the models used here. These data were sourced from the statistics of air transport from the Ministry of Land, Infrastructure, Transport and Tourism, Japan (MLIT) [28].

### Meteorological data

Meteorological conditions such as average temperature (unit: °C) and relative humidity (unit: %) are thought to be significantly associated with the occurrence of the HRSV epidemic in the temperate regions of Japan [26]. Therefore, we used these meteorological data (monthly average temperature and relative humidity) published by the Japan Meteorological Agency (JMA) as explanatory variables in the models presented here [29]. Monthly meteorological data collected from meteorological observatories situated in the prefectural capital city were used for each prefecture. We extracted the monthly average temperature and relative humidity for the 7 epidemiologic years (2014–2020). There were two meteorological observatories with missing data on relative humidity. Therefore, we selected observatories at the nearest distance (≤50 km) from the prefectural capital by substitution. Using the monthly meteorological data for each prefecture, the average temperature and relative humidity over the whole of Japan were calculated.

### Statistical testing and modeling approach

First, we conducted a descriptive analysis of the study period (2014–2020) to identify the characteristics of the dataset included in this study. Specifically, we visualized time-series seasonal variations in the number of HRSV cases per sentinel site (HRSV activity), NPI indicators (retail sales of hand hygiene products, and the number of international and domestic airline passenger arrivals), and meteorological conditions (average temperature and relative humidity) during the study period. We then compared monthly HRSV activity in 2020 with the average HRSV activity in the corresponding period in the 6 preceding epidemiologic years (January 2014–December 2020) using a monthly paired *t*-test.

In the construction of the predictive model, several steps were taken to build a robust and reliable model. Prior to constructing the model, we checked the probability distribution of the monthly number of HRSV cases per sentinel site at the national level (normality examined by the Shapiro-Wilk test) (Additional file 1: Figure S1) and assessed the linearity between HRSV activity with each independent variable. We adopted a generalized linear regression model (GLM) with gamma distribution and log link function, allowing for overdispersion, to investigate the association between monthly HRSV activity and NPIs (retail sales of hand hygiene products and the number of international and domestic airline passenger arrivals), while adjusting for meteorological conditions (monthly average temperature and relative humidity) and seasonal variations. The monthly number of HRSV cases per sentinel site (continuous) was the dependent variable, and monthly retail sales of hand hygiene products per ¥1 billion (continuous), monthly number of international and domestic airline passenger arrivals per 100,000 population (continuous), and meteorological conditions (monthly average temperature and relative humidity) (continuous) were included as explanatory variables. Furthermore, the model was adjusted by using year variables (2014, 2015, 2016, 2017, 2018, 2019, and 2020) (category) as a covariate to control seasonal variation. The goodness-of-fit of the predictive model was assessed in a combined way using the dispersion parameter (*α*) and the Akaike Information Criterion (AIC) [30, 31]. *α* is the variance parameter of the model, an *α* value of less than 1.5, suggests that the deviation of the observed data from the model is not too large (i.e., the model fits the observed data well). Although there is no theoretical basis for this criterion, it has been shown that *α* < 1.5 can significantly improve the degree of overdispersion [31, 32]. In the present analysis, the overdispersion of each model was mitigated using gamma distribution to ensure that *α* was less than 1.5. Generally, the best model with a lower AIC value is preferred as it achieves a more optimal combination of goodness-of-fit and parsimony. AIC was calculated as –2*ln*(*L*) + 2 *K* where *ln*(*L*) is the maximum value of the log-likelihood function of the predictive model and *k* represents the number of parameters.

All explanatory variables included in the predictive model were examined for multicollinearity using Spearman’s rank-order correlation coefficient (*ρ*). If the variables were found to be highly correlated (|*ρ*| > 0.8) [33], we selected the variable with the strongest statistical correlation with the dependent variable and discarded the other variables. In the present analysis, no variables showing a strong correlation were observed (Additional file 1: Table S1).

Additionally, we considered lags (delays in effect) of up to 4 months from several previous studies [34–40]. Specifically, Spearman’s rank-order correlation coefficients between the monthly number of HRSV cases per sentinel site and the retail sales of hand hygiene products, the number of international and domestic airline passenger arrivals, and meteorological conditions (monthly average temperature and relative humidity) with lags of 0–4 months were considered. Since including all relevant lags in the model could lead to significant collinearity, a new explanatory variable was created using the moving averages of the two lags that had the greatest significant correlation coefficient and were incorporated into the final predictive model [41, 42]. Based on analysis results, we created new explanatory variables: moving average of retail sales of hand hygiene products for the current month and to 1 month (lag 0–1 months average), moving average of domestic airline passengers and international airline passenger arrivals for 1 month and 2 months (lag 1–2 months average), moving average of average temperature for 3 months and 4 months (lag 3–4 months average), and moving average of relative humidity for 1 month and 2 months (lag 1–2 months average), and developed a final predictive model that takes these into account (Additional file 1: Table S2).

In summary, the predictive model was conceptualized using the following formula:

1 $$ {Y}_t\mid {E}_Y\sim Gamma\left({E}_Y\right) $$

2 $$ \mathit{\ln}\left({E}_Y\right):= {\beta}_1f\left( NPI{s}_t\right)+{\beta}_2f\left({T}_t\right)+{\beta}_3f\left(R{H}_t\right)+ Yea{r}_i+{\eta}_i $$

where *Y*_*t*_ is the outcome series and *E*_*Y*_ is the expected time-series of the monthly HRSV activity. *f(NPI*_*t*_*)* is the moving average of the two lags that had the greatest significant correlation coefficient of monthly NPI indicators (retail sales of hand hygiene products, the number of international and domestic passenger arrivals) with HRSV activity with a lag of 0–4 months; *f*(*T*_*t*_) and *f*(*RH*_*t*_) indicate moving averages of the two lags that had the greatest significant correlation coefficient of monthly average temperature and relative humidity, respectively, with HRSV activity with a lag of 0–4 months. The terms *β*_*1*_, *β*_*2*_, and *β*_*3*_ indicate regression coefficients, *Year*_*i*_ represents indicator variables of year, and term *η*_*i*_ corresponds to the intercept.

Four final predictive models were constructed to evaluate the direct effects of each NPI indicators. Model 0 is a base model adjusted for meteorological and year variables. Models 1, 2, and 3 used the same variables as Model 0, plus the retail sales of hand hygiene products, the number of domestic airline passenger arrivals, and the number of international airline passenger arrivals, respectively. Regression coefficients and *p*-values were calculated for each predictive model. To examine the robustness of the main findings, after establishing the final model using the process outlined above, we performed sensitivity analyses for the main analyses with a long lag period for each NPI indicator, depending on the lag effect selection. Statistical significance was set at *p*-value of less than 0.05, on a two-tailed test. All analyses were performed using STATA 15.1 software (Stata Corp, College Station, TX, USA).

### Ethical considerations

The present ecological study analyzed publicly available data. As such, the datasets used in our study were de-identified and fully anonymized in advance, and the analysis of publicly available data without any identifiable information does not require ethical approval.

### Data sources

An anonymized dataset that enables the replication of the analysis are publicly available (sources are mentioned in Methods and References). Datasets generated during the study are available on request from the corresponding author.

## Results

### Long-term monthly seasonal variations in HRSV activity, NPI indicators, and meteorological conditions during 2014–2020

**Fig. 1:**
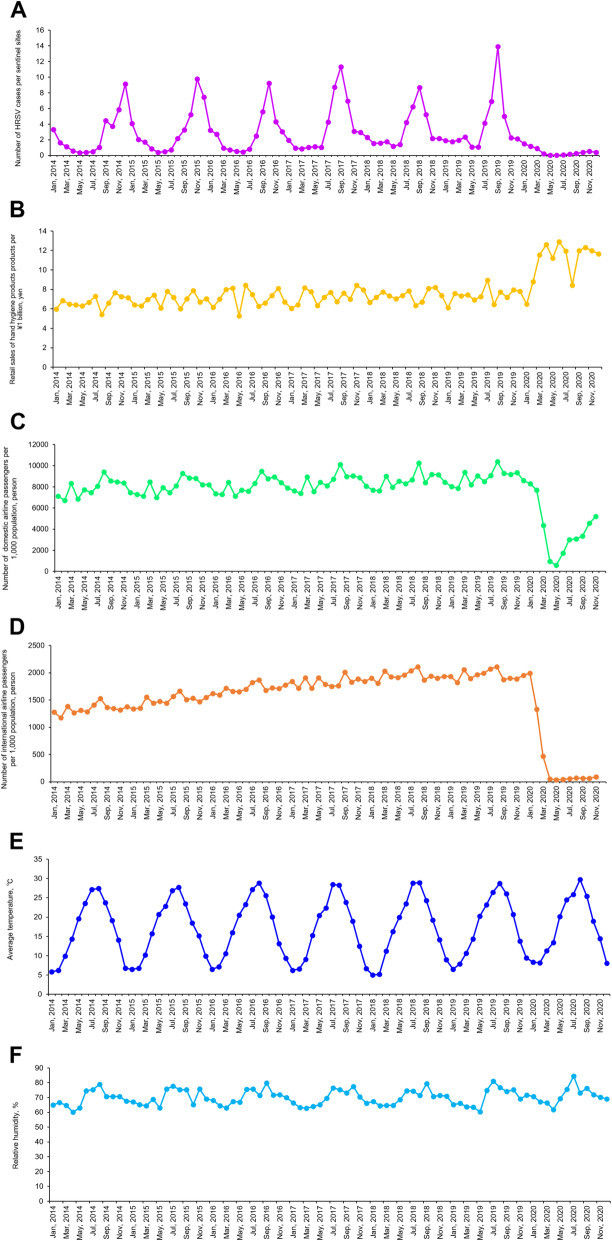
Monthly seasonal variations of number of HRSV activity, NPI indicators, and meteorological conditions during 2014–2020. **a** Monthly seasonal variations of number of HRSV cases per sentinel sites based on national HRSV surveillance data during 2014–2020. **b** Monthly seasonal variations of retail sales of hand hygiene products per ¥1 billion (unit: yen) during 2014–2020. **c** Monthly seasonal variations of number of domestic airline passengers per 1000 population (unit: person) during 2014–2020. **d** Monthly seasonal variations of number of international airline passengers per 1000 population (unit: person) during 2014–2020. **e** Monthly seasonal variations of average temperature (unit: °C) throughout Japan during 2014–2020. **f** Monthly seasonal variations of relative humidity (unit: %) throughout Japan during 2014–2020

Figure 1a shows the monthly number of HRSV cases per sentinel site (HRSV activity) in Japan for the 7 epidemiologic years (2014–2020), illustrating the decrease in HRSV activity during the COVID-19 pandemic in 2020. Typically, HRSV follows a seasonal pattern, with HRSV cases peaking in the winter months (November–March) in temperate regions, such as Japan. The average monthly HRSV activity was relatively similar each year: 3.69 cases in 2019, 3.19 cases in 2018, 3.68 cases in 2017, 2.83 cases in 2016, 3.18 cases in 2015, and 2.66 cases in 2014; however 2020 showed the lowest incidence with 0.47 cases. Specifically, the average number of HRSV cases during January–December 2020 decreased by approximately 85% (paired *t*-test, *p* < 0.001) compared to those in the preceding 6 epidemiologic years (2014–2019). In addition, the monthly HRSV activity during the COVID-19 pandemic in Japan dramatically decreased from January to May 2020, with no major epidemic peaks observed in contrast to the previous years.

Monthly variation was observed in the retail sales of hand hygiene products, the number of international and domestic airline passenger arrivals, and meteorological conditions (monthly average temperature and relative humidity) during the study period (2014–2020) (Fig. 1b-f). The monthly retail sales of hand hygiene products, shown in Fig. 1b, has remained constant at approximately ¥7 billion (approximately £47,600,000/$63,000,000) for the past 6 years (2014–2019), but a sharp upward trend was observed in January 2020. Specifically, there was a sharp increase between January and April 2020, a slight decrease in May, before an increase again in June and July which was sustained at approximately ¥11 billion (approximately £74,800,000/$99,000,000). Monthly average number of domestic and international passenger arrivals was approximately 8.3 million and 1.7 million people respectively for the past 6 years, which declined sharply from January to May 2020. (Fig. 1c and d). In 2020, the number of both domestic and international airline passengers was low, but the former showed recovery from May onwards. The average temperature and relative humidity did not vary significantly throughout the study period (Fig. 1e and f). The average temperature peaks at approximately 27 °C in August of each year and the relative humidity was approximately 60–80% throughout the year.

### Association between monthly HRSV activity and NPI indicators adjusted for meteorological conditions and seasonality during 2014–2020

Results of the time-series generalized linear gamma regression analysis for monthly HRSV activity are shown in Table 1. Regression analysis showed that retail sales of hand hygiene products and the number of international and domestic passenger arrivals were significantly associated with monthly HRSV activity after adjusting for meteorological conditions (monthly average temperature and relative humidity) and year variables (seasonality) during 2014–2020 (Table 1). We found that every average ¥1 billion increase spent on retail hand hygiene products during the current month and one month before (lag 0–1 months) was independently associated with a 0.29% decrease in the monthly HRSV activity (Model 1: coefficient − 0.29, *p* = 0.003, *α* = 0.47). In contrast, in Models 2 and 3, an increase of an average 1000 domestic and international airline passenger arrivals during the 1 through the 2 months before (lag 1–2 months) was associated with a 3.8 × 10^− 4^% and 1.2 × 10^− 3^% increase in monthly HRSV activity, respectively (Model 2: coefficient 3.8 × 10^− 4^, *p* < 0.001, *α* = 0.30; Model 3: coefficient 1.2 × 10^− 3^, *p* < 0.001, *α* = 0.32). For the year variable in each model, 2020 was associated with a greater decrease in HRSV activity than in the other years (2014–2019).

**Table 1:** Regression coefficient for HRSV activity: the results of a multivariate analysis

		Model 0^b^		Model 1^c^		Model 2^d^		Model 3^e^	
Variable	Lag (months)^a^	Coef.	*p*	Coef.	*p*	Coef.	*p*	Coef.	*p*
NPI indicator									
Retail sales of hand hygiene products^f^	0–1			−0.29	0.003				
Domestic airline passenger arrivals^g^	1–2					3.8 × 10^− 4^	< 0.001		
International airline passenger arrivals^h^	1–2							1.2 × 10^−3^	< 0.001
Other covariates									
Average temperature^i^	3–4	0.06	< 0.001	0.06	< 0.001	0.04	0.001	0.07	< 0.001
Relative humidity^j^	1–2	0.05	0.06	0.04	0.08	0.05	0.007	0.05	0.02
Intercept		−4.26	0.01	−1.89	0.32	−7.17	< 0.001	−5.82	< 0.001
Year									
2014		Ref.		Ref.		Ref.		Ref.	
2015		0.20	0.52	0.20	0.51	0.24	0.33	0.09	0.72
2016		0.13	0.96	0.05	0.86	−0.02	0.91	−0.37	0.16
2017		0.49	0.13	0.60	0.06	0.30	0.24	−0.03	0.90
2018		0.47	0.16	0.57	0.08	0.24	0.34	−0.23	0.44
2019		0.53	0.11	0.68	0.04	0.18	0.48	−0.20	0.49
2020		−1.55	< 0.001	−0.63	0.19	−0.66	0.04	−1.07	0.001
Model statistics									
*α*		0.50		0.47		0.30		0.32	
AIC		3.63		3.60		3.47		3.50	

*Abbreviations*: *Coef.* regression coefficient, *se* standard deviation, *AIC* Akaike information criterion, *α* dispersion parameter

^a^ Moving average

^b^ Adjusted for monthly average temperature at a lag of 3–4 months, monthly relative humidity at lag a of 1–2 months, and year variables (2014, 2015, 2016, 2017, 2018, 2019, and 2020)

^c^ Generalized linear gamma regression model adjusted for the monthly retail sales of hand hygiene products per ¥1 billion at a lag of 0–1 months, monthly average temperature at a lag of 3–4 months, monthly relative humidity at a lag of 1–2 months, and year variables (2014, 2015, 2016, 2017, 2018, 2019, and 2020)

^d^ Generalized linear gamma regression model adjusted for monthly number of domestic airline passenger arrivals per 1000 population at a lag of 1–2 months, monthly average temperature at a lag of 3–4 months, monthly relative humidity at a lag of 1–2 months, and year variables (2014, 2015, 2016, 2017, 2018, 2019, and 2020)

^e^ Generalized linear gamma regression model adjusted for number of international airline passenger arrivals per 1000 population at a lag of 1–2 months, monthly average temperature at a lag of 3–4 months, monthly relative humidity at a lag of 1–2 months, and year variables (2014, 2015, 2016, 2017, 2018, 2019, and 2020)

^f^ Retail sales of hand hygiene products per ¥1 billion (unit: yen)

^g^ Number of domestic airline passenger arrivals per 1000 population (unit: person)

^h^ Number of international airline passenger arrivals per 1000 population (unit: person)

^i^ Average temperature (unit: °C)

^j^ Relative humidity (unit: %)

### Further investigations

We conducted sensitivity analyses to verify the robustness of our results. Specifically, we varied the lag time of each NPI indicator for a longer period and performed the same analysis outlined above. The results suggested that the long lag period for the retail sales of hand hygiene products was associated with a greater reduction in the HRSV activity compared to the main analysis (Additional file 1: Table S3). On the other hand, a longer lag period for domestic and international airline passenger arrivals was associated with a slightly smaller decrease in HRSV activity compared to the main analysis (Additional file 1: Tables S4–5). These sensitivity analyses confirm the robustness of the main analysis and our main findings were largely insensitive.

## Discussion

In the present study, we assessed the association between HRSV activity and NPI indicators such as sanitary measures and travel restrictions, focusing on the acute effects of the COVID-19 pandemic. Despite the simplified assumptions, our results suggest that the NPIs in Japan may have had a positive impact on the transmission dynamics of HRSV. Specifically, the average monthly HRSV cases (HRSV activity) in 2020 decreased by approximately 85% compared with that of the previous 6 years (2014–2019). Notably, reductions in HRSV activity of 0.29%, 3.8 × 10^− 4^%, and 1.2 × 10^− 3^% were independently associated with an increase per unit in the amount spent on retail sales of hand hygiene products at lag of 0–1 months and a decrease per unit in the number of domestic and international passenger arrivals at lag of 1–2 months, respectively. In 2020, we found a significant decrease in HRSV activity across multiple NPI indicators. Additionally, our results suggest that there may be a long-term temporal association between HRSV activity and NPIs, i.e., the effect of these NPIs on HRSV activity does not necessarily mean that they had an effect only within this follow-up period (2014–2020). Our results support our hypothesis that sanitary measures (such as hand hygiene products) and travel restrictions imposed to control the COVID-19 pandemic may have potentially reduced the HRSV transmissibility and provide further insights into their public health benefits.

To our knowledge, studies investigating the association between HRSV activity and NPI indicators, including the sale of retail of hand hygiene products and international and domestic airline passengers during the COVID-19 pandemic, have been scarce to date. However, Cowling et al. reported that various NPIs used during the COVID-19 pandemic in Hong Kong may have had a significant effect on the seasonal influenza epidemic [43]. Specifically, seasonal influenza transmission decreased significantly after social distancing measures were implemented and population behavior changed, with the rate of infections in the community decreasing by 44%. They also reported that 85% of respondents avoided crowds and 99% said they wore a face mask when they went out during the study period. A study including two hospitals in Finland showed a significant reduction in seasonal influenza and HRSV cases in children after a national lockdown, suggesting an immediate effect of social distancing [44]. Similarly, in Japan, it has been suggested that widespread promotion of personal protective measures, such as wearing masks and hand washing, since the beginning of the epidemic may have significantly reduced the transmissibility of seasonal influenza [45–48]. Based on the above effects of NPIs, we speculate that the Japanese government’s NPI strategy may have also contributed to reducing the HRSV transmission. The reasons for the reduction in HRSV transmission observed in this study were multifactorial, as it was difficult to isolate the effects of sanitary measures and travel restrictions, which were examined in this study. These NPI strategies are effective in reducing viral transmission in the community, but convincing evidence to support their effectiveness is currently lacking. Further detailed long-term studies are needed to clarify the causal relationships and complex interactions.

The implementation of hand hygiene strategies, which is feasible and recommended for use in a variety of settings, is one of the interventions that can contribute to the achievement of sustainable development goals (SDGs) [49, 50]. These interventions have been introduced in various countries and are considered an important strategy. Two previous systematic reviews and meta-analyses of local communities in low-income and middle-income countries have estimated that hand hygiene may reduce the transmission of respiratory infections by approximately 16 and 21%, respectively [51, 52]. Unlike for seasonal influenza, no licensed prophylactic vaccine has been developed and supplied to date for HRSV. With regards to drugs targeted against HRSV, palivizumab (anti-HRSV antibody), the humanized monoclonal antibody to HRSV fusion (F) protein, is currently limited to passive vaccination for the prevention of severe disease [2, 53, 54]. Therefore, as a vaccine has not yet been developed, thorough hand hygiene can be an important intervention to reduce person-to-person transmission of HRSV, thus preventing an epidemic. Importantly, our preliminary findings suggest a possible contribution of sanitary measures to the reduction of HRSV activity in Japan; however, no information is available for other regions or countries. The effects of hand hygiene in other regions and countries with different socio-economic statuses may differ from those in Japan [55, 56]. Recognizing the importance of hand hygiene, timely surveillance, and further detailed studies at individual- and community-level are needed to assess the differences in the effectiveness after the COVID-19 pandemic in Japan.

It is important to note that in the present study, we focused on the association between human mobility, such as domestic and international airline passengers, and the transmission dynamics of HRSV. In particular, a major shift in the occurrence of HRSV has been observed in Japan since around 2016 [26]; although there are reports suggesting a possible association with international passengers [57, 58], there are currently no reports directly assessing this. The effect of the COVID-19 pandemic has led to a number of previous studies highlighting the effect of international travel restrictions on COVID-19 activity [59–63], providing an excellent opportunity to consider the effect of human mobility on other respiratory infectious diseases. Especially in small island countries such as Japan, quarantine at a border (i.e., travel restrictions) may contribute significantly to preventing the arrival of the virus (or at least delay the arrival) [64]. However, there are a limited number of studies reporting the effects of domestic travel restrictions on the virus spread. A recently published report by Murano et al., quantifying the effect of domestic travel restrictions and COVID-19 spread in Japan, provides interesting insights into the transmission dynamics of other respiratory infections, including HRSV [65]. Specifically, transmission dynamics varied in the degree of reduction in the number of passengers and the centrality of each prefecture in the public transport network (e.g., car, train, ship, airline network), assuming that traffic restrictions were in place. The relative risk reduction in most prefectures (approximately 35–48%), as reported by Murano et al., suggests that the degree of passenger volume has a significant effect on risk reduction and concluded that the optimal size of the reduction may depend strongly on the domestic network. In our study, both international and domestic travel contributed to a significant reduction in HRSV activity after adjustment for other covariates. Strategies to restrict the airline network may reduce the risk of HRSV transmission at certain risk-sensitive airports. This study provides an important perspective on how restrictions on international and domestic human mobility may affect HRSV activity.

We have noted that the transmission dynamics of HRSV in Japan in 2020 were very different from previous years, and would like to add that similar trends (i.e., decline) in other infectious and non-infectious respiratory diseases have been reported in many countries, including Japan, Brazil, Pakistan, Germany, Taiwan, Singapore, and China [17–21, 66–74]. In these regions, in addition to HRSV and seasonal influenza, tuberculosis, rubella, norovirus, pertussis, diphtheria, Epstein-Barr virus, pneumonia, and influenza mumps have been reported to be reduced by an average of ≥70%, suggesting a contribution of NPIs to these phenomena [17–21, 66–74]. It is important to quantify the impact of NPIs on the transmission dynamics of infectious and non-infectious respiratory diseases in the future.

The findings of this study should be interpreted in the context of several limitations. First, it should be noted that these are preliminary findings describing the acute effects of NPIs on HRSV activity in Japan, and this ecological analysis cannot prove a causal relationship. Second, this study used retail sales of hand hygiene products and domestic and international airline passengers as alternative indicators of NPIs using published data available in Japan. Therefore, it is possible that these alternative indicators do not reflect the actual effects of NPIs. Third, the present study did not examine the effect of combining multiple strategies of NPIs on HRSV (e.g., effect modification by interaction term), and reports the independent effect of NPIs. Generally, it has been suggested that the effectiveness of NPIs may increase when multiple strategies are combined [39, 40]. Further detailed studies are needed to independently evaluate the effects of individual and multiple interventions and their complementarity on host susceptibility. Fourth, several other NPIs were not considered in this study, such as education, voluntary isolation, school closure, and voluntary mask wearing [75–78]. Cultural factors, such as social habits and family size, may influence the transmission of HRSV [79]. Other factors, such as the emergence of new viruses, the viral interference (i.e., inhibition of growth of one virus by another), and the development of immunity during the study period were not assessed. In particular, a new HRSV type A, ON1, has emerged and circulated worldwide in recent years [23, 80–82]. The emergence of such strains may affect the timing of HRSV epidemics. Furthermore, it has been suggested that the recent increase in reported cases of HRSV infections in Japan may be due to the increased frequency of testing and the expansion of insurance coverage for HRSV testing in late 2011. This may have influenced the true increase in HRSV activity in Japan. Indeed, the HRSV transmission dynamics may be obscured and regulated by these potential intrinsic factors, and detailed studies are needed to assess the direct effects of these factors. In particular, it would be useful to examine and analyze the effects of other specific NPIs (e.g., voluntary mask wearing and school closure) in sentinel sites where the data was collected. In this present study, we were unable to include these factors due to the lack of published available data relating to voluntary mask wearing and school closures at each sentinel site, as well as the considerable variation in the duration of coverage between prefectures for school closures. Further studies aimed at a prefectural-level are needed to quantify the extent to which these factors contribute to the transmission dynamics of HRSV between prefectures. Fifth, it is possible that the decline observed in Japan in 2020 was only the natural end of the HRSV epidemic. However, if we review the epidemic dynamics in Japan in previous years, the change in the rate of decline after 2020 is dramatic, suggesting that other factors are involved. Sixth, although average temperature and relative humidity were used as meteorological conditions in the present analysis, there may be other confounding factors that could be associated with HRSV activity (e.g., rainfall, wind speed, and air pollutants) [83, 84]. However, previous studies have suggested a consistent and strong association between HRSV activity and average temperature/relative humidity, which may have been a predictor with high explanatory power [35, 85]. Seventh, because the present study focused on the transmission dynamics of HRSV nationwide in Japan, it was difficult to detect differences in the incidence of HRSV in specific regions during study period (2014–2020). However, our recent study that uses prefectural-level data showed that an earlier onset week of the HRSV epidemic season in Japan was associated with an increased number of inbound overseas travelers, supporting our present findings [58]. This previous research covers the period before the COVID-19 pandemic (2014–2017) and, in addition, does not take into account the effect of retail sales of hand hygiene products, which critically needs further detailed studies at the prefectural-level in Japan in post-pandemic period. Eighth, we were also unable to fully assess the association between the incidence of HRSV in specific regions during a downturn in retail sales of hand hygiene products or rebound in domestic travel because our study focused on Japan nationwide, instead of individual prefectures. Indeed, small outbreaks were observed in some of Japans’ southernmost prefectures, such as Okinawa in November and Kagoshima in October 2020, following the decline in retail sales of hand hygiene products that occurred around August 2020 and rebound in domestic travel that began around July 2020 [58]. However, no outbreaks of HRSV were observed in the rest of the country in Japan. Therefore, to identify the actual causes of these heterogeneous local outbreaks, it is necessary to consider a more detailed time-series analysis for each NPIs indicator at the prefectural-level in Japan. Finally, the epidemiological effects of NPIs have not yet been adequately quantified. For instance, the increase in local clusters of COVID-19 in remote prefectures in Japan should be considered [86–88], and the epidemiological impact of NPIs on HRSV activity may be different (e.g., less effect of NPIs).

## Conclusions

Despite the number of limitations noted above, our study showed a possible association between more NPI indicators such as sanitary measures and travel restrictions, and decreased HRSV activity during the COVID-19 pandemic in Japan, suggesting that the hypothesis that these public health interventions for COVID-19 adopted by the Japanese government may have prevented and suppressed the spread of HRSV. Furthermore, we believe that the present study provides critical insights into the epidemiological impact of NPIs on the transmission dynamics of HRSV in Japan. The decline in HRSV cases in Japan appears to be concurrent with the COVID-19 pandemic and related community public health measures. The current preliminary findings reaffirm the usefulness of the NPI measures recommended by WHO and remind us of the need and importance of educational campaigns and the practical implementation of public health policies. In particular, with a continuous circulation of COVID-19 and use of NPIs in many countries, detailed studies are critically needed to evaluate the causes of decreased respiratory infections [89–91]. Additionally, until viable pharmaceutical options for HRSV become available, it is important to continue to evaluate non-pharmaceutical strategies and their potential benefits. Accordingly, assessing the future transmission dynamics and risks of HRSV and other respiratory infections, taking into account the detailed effects of various public health interventions, will help guide the design of future broader infectious disease control measures and provide critical lessons for other countries.

## Supplementary Information

**Additional file 1 **: **Figure S1.** Probability distribution of monthly HRSV activity at national level in Japan during 2014–2020. **Table S1.** Spearman’s rank-order correlation coefficients matrix of monthly variables included in this study during 2014–2020. **Table S2.** Spearman’s rank-order correlation coefficients showing the lag effect for individual NPI indicators and meteorological conditions. **Table S3.** Regression coefficient for HRSV activity: the results of a multivariate analysis. **Table S4.** Regression coefficient for HRSV activity: the results of a multivariate analysis. **Table S5.** Regression coefficient for HRSV activity: the results of a multivariate analysis.

## Data Availability

An anonymized dataset that enables the replication of the analysis are publicly available (sources are mentioned in Methods and References). Datasets generated during the study are available on request from the corresponding author.

## Abbreviations

HRSV: Human respiratory syncytial virus
ALRIs: Acute lower respiratory tract infections
COVID-19: Coronavirus disease 2019
SARS-CoV-2: Severe acute respiratory syndrome coronavirus 2
NPIs: Non-pharmaceutical interventions
WHO: World Health Organization
IDWR: Infectious Disease Weekly Report
NESID: National Epidemiological Surveillance of Infectious Diseases
NIID: National Institute of Infectious Diseases, Japan
MHLW: Ministry of Health, Labor and Welfare, Japan
RDT: Rapid diagnostic test
METI: Ministry of Economy, Trade and Industry, Japan
MLIT: Ministry of Land, Infrastructure, Transport and Tourism, Japan
JMA: Japan Meteorological Agency
GLM: Generalized linear model
AIC: Akaike Information Criterion
SDGs: sustainable development goals

## References

[1] Piedimonte G, Perez MK (2014). Respiratory syncytial virus infection and bronchiolitis. Pediatr Rev.

[2] Nair H, Nokes DJ, Gessner BD, Dherani M, Madhi SA, Singleton RJ, O'Brien KL, Roca A, Wright PF, Bruce N (2010). Global burden of acute lower respiratory infections due to respiratory syncytial virus in young children: a systematic review and meta-analysis. Lancet (London, England).

[3] Hirve S, Crawford N, Palekar R, Zhang W (2020). Clinical characteristics, predictors, and performance of case definition-interim results from the WHO global respiratory syncytial virus surveillance pilot. Influenza Other Respir Viruses.

[4] Wang X, Li Y, Deloria-Knoll M, Madhi SA, Cohen C, Ali A, Basnet S, Bassat Q, Brooks WA, Chittaganpitch M, Echavarria M, Fasce RA, Goswami D, Hirve S, Homaira N, Howie SRC, Kotloff KL, Khuri-Bulos N, Krishnan A, Lucero MG, Lupisan S, Mira-Iglesias A, Moore DP, Moraleda C, Nunes M, Oshitani H, Owor BE, Polack FP, O'Brien KL, Rasmussen ZA, Rath BA, Salimi V, Scott JAG, Simões EAF, Strand TA, Thea DM, Treurnicht FK, Vaccari LC, Yoshida LM, Zar HJ, Campbell H, Nair H, Libster R, Otieno G, Joundi I, Broor S, Nicol M, Amarchand R, Shi T, López-Labrador FX, Baker JM, Jamison A, Choudekar A, Juvekar S, Obermeier P, Schweiger B, Madrid L, Thomas E, Lanaspa M, Nohynek H, Nokes J, Werner M, Danhg A, Chadha M, Puig-Barberà J, Caballero MT, Mathisen M, Walaza S, Hellferscee O, Laubscher M, Higdon MM, Haddix M, Sawatwong P, Baggett HC, Seidenberg P, Mwanayanda L, Antonio M, Ebruke BE, Adams T, Rahman M, Rahman MZ, Sow SO, Baillie VL, Workman L, Toizumi M, Tapia MD, Nguyen T, Morpeth S (2021). Global burden of acute lower respiratory infection associated with human metapneumovirus in children under 5 years in 2018: a systematic review and modelling study. Lancet Glob Health.

[5] Mazur NI, Higgins D, Nunes MC, Melero JA, Langedijk AC, Horsley N, Buchholz UJ, Openshaw PJ, McLellan JS, Englund JA (2018). The respiratory syncytial virus vaccine landscape: lessons from the graveyard and promising candidates. Lancet Infect Dis.

[6] Heymann DL, Shindo N (2020). COVID-19: what is next for public health?. Lancet (London, England).

[7] Wang C, Horby PW, Hayden FG, Gao GF (2020). A novel coronavirus outbreak of global health concern. Lancet (London, England).

[8] World Health Organization (WHO) (2021). Coronavirus disease 2019 (COVID-19) Weekly Epidemiological Update and Weekly Operational Update.

[9] Ministry of Health, Labour and Welfare, Japan (MHLW) (2021). Coronavirus disease 2019 (COVID-19) domestic outbreaks.

[10] Flaxman S, Mishra S, Gandy A, Unwin HJT, Mellan TA, Coupland H, Whittaker C, Zhu H, Berah T, Eaton JW, Monod M, Perez-Guzman PN, Schmit N, Cilloni L, Ainslie KEC, Baguelin M, Boonyasiri A, Boyd O, Cattarino L, Cooper LV, Cucunubá Z, Cuomo-Dannenburg G, Dighe A, Djaafara B, Dorigatti I, van Elsland SL, FitzJohn RG, Gaythorpe KAM, Geidelberg L, Grassly NC, Green WD, Hallett T, Hamlet A, Hinsley W, Jeffrey B, Knock E, Laydon DJ, Nedjati-Gilani G, Nouvellet P, Parag KV, Siveroni I, Thompson HA, Verity R, Volz E, Walters CE, Wang H, Wang Y, Watson OJ, Winskill P, Xi X, Walker PGT, Ghani AC, Donnelly CA, Riley S, Vollmer MAC, Ferguson NM, Okell LC, Bhatt S, Imperial College COVID-19 Response Team (2020). Estimating the effects of non-pharmaceutical interventions on COVID-19 in Europe. Nature.

[11] Lai S, Ruktanonchai NW, Zhou L, Prosper O, Luo W, Floyd JR, Wesolowski A, Santillana M, Zhang C, Du X (2020). Effect of non-pharmaceutical interventions to contain COVID-19 in China. Nature.

[12] Anderson RM, Heesterbeek H, Klinkenberg D, Hollingsworth TD (2020). How will country-based mitigation measures influence the course of the COVID-19 epidemic?. Lancet (London, England).

[13] Haug N, Geyrhofer L, Londei A, Dervic E, Desvars-Larrive A, Loreto V, Pinior B, Thurner S, Klimek P (2020). Ranking the effectiveness of worldwide COVID-19 government interventions. Nat Hum Behav.

[14] World Health Organization (WHO) Regional Office for the Western Pacific (WPRO) (2020). Calibrating long-term non-pharmaceutical interventions for COVID-19: principles and facilitation tools.

[15] World Health Organization (WHO) (2021). Coronavirus disease 2019 (COVID-19) advice for the public.

[16] Baker RE, Park SW, Yang W, Vecchi GA, Metcalf CJE, Grenfell BT (2020). The impact of COVID-19 nonpharmaceutical interventions on the future dynamics of endemic infections. Proc Natl Acad Sci U S A.

[17] Friedrich F, Ongaratto R, Scotta MC, Veras TN, Stein R, Lumertz MS, et al. Early impact of social distancing in response to COVID-19 on hospitalizations for acute bronchiolitis in infants in Brazil. Clin Infect Dis. 2021;72(12):2071–5. 10.1093/cid/ciaa1458. 10.1093/cid/ciaa1458 PMC7543304 32986818

[18] Soo RJJ, Chiew CJ, Ma S, Pung R, Lee V (2020). Decreased influenza incidence under COVID-19 control measures, Singapore. Emerg Infect Dis.

[19] Kuo SC, Shih SM, Chien LH, Hsiung CA (2020). Collateral benefit of COVID-19 control measures on influenza activity, Taiwan. Emerg Infect Dis.

[20] Sakamoto H, Ishikane M, Ueda P (2020). Seasonal influenza activity during the SARS-CoV-2 outbreak in Japan. Jama.

[21] Olsen SJ, Azziz-Baumgartner E, Budd AP, Brammer L, Sullivan S, Pineda RF, Cohen C, Fry AM (2020). Decreased influenza activity during the COVID-19 pandemic - United States, Australia, Chile, and South Africa, 2020. MMWR Morb Mortal Wkly Rep.

[22] National Institute of Infectious Diseases, Japan (NIID) (2021). National Epidemiological Surveillance of Infectious Diseases (NESID) Infectious Diseases Weekly Report (IDWR).

[23] Hibino A, Saito R, Taniguchi K, Zaraket H, Shobugawa Y, Matsui T, Suzuki H (2018). Molecular epidemiology of human respiratory syncytial virus among children in Japan during three seasons and hospitalization risk of genotype ON1. PLoS One.

[24] Zaraket H, Saito R (2016). Japanese surveillance systems and treatment for influenza. Curr Treat Options Infect Dis.

[25] Ministry of Health, Labour and Welfare, Japan (MHLW) (2020). Notification of physicians and veterinarians based on the infectious diseases law (human respiratory syncytial virus).

[26] Shobugawa Y, Takeuchi T, Hibino A, Hassan MR, Yagami R, Kondo H, Odagiri T, Saito R (2017). Occurrence of human respiratory syncytial virus in summer in Japan. Epidemiol Infect.

[27] Ministry of Economy, Trade and Industry, Japan (METI) (2021). Statistics of Production of Chemical Industry of the Ministry of Economy, Trade and Industry, Japan.

[28] Ministry of Land, Infrastructure, Transport and Tourism, Japan (MLIT) (2021). Statistics of Air Transport of the Ministry of Land, Infrastructure, Transport and Tourism, Japan.

[29] Japan Meteorological Agency (JMA) (2021). Meteorological Data Search.

[30] Akaike H (1974). A new look at the statistical model identification. IEEE Trans Autom Control.

[31] McCullagh P (1984). Generalized linear models. Eur J Oper Res.

[32] Ohkubo Y, Yamamoto T, Ogusu N, Watanabe S, Murakami Y, Yagi N, Hasegawa E (2018). The benefits of grouping as a main driver of social evolution in a halictine bee. Sci Adv.

[33] Chan YH (2003). Biostatistics 104: correlational analysis. Singap Med J.

[34] Tang JW, Lai FY, Wong F, Hon KL (2010). Incidence of common respiratory viral infections related to climate factors in hospitalized children in Hong Kong. Epidemiol Infect.

[35] Thongpan I, Vongpunsawad S, Poovorawan Y (2020). Respiratory syncytial virus infection trend is associated with meteorological factors. Sci Rep.

[36] Li Y, Reeves RM, Wang X, Bassat Q, Brooks WA, Cohen C, Moore DP, Nunes M, Rath B, Campbell H, Nair H, Acacio S, Alonso WJ, Antonio M, Ayora Talavera G, Badarch D, Baillie VL, Barrera-Badillo G, Bigogo G, Broor S, Bruden D, Buchy P, Byass P, Chipeta J, Clara W, Dang DA, de Freitas Lázaro Emediato CC, de Jong M, Díaz-Quiñonez JA, Do LAH, Fasce RA, Feng L, Ferson MJ, Gentile A, Gessner BD, Goswami D, Goyet S, Grijalva CG, Halasa N, Hellferscee O, Hessong D, Homaira N, Jara J, Kahn K, Khuri-Bulos N, Kotloff KL, Lanata CF, Lopez O, Lopez Bolaños MR, Lucero MG, Lucion F, Lupisan SP, Madhi SA, Mekgoe O, Moraleda C, Moyes J, Mulholland K, Munywoki PK, Naby F, Nguyen TH, Nicol MP, Nokes DJ, Noyola DE, Onozuka D, Palani N, Poovorawan Y, Rahman M, Ramaekers K, Romero C, Schlaudecker EP, Schweiger B, Seidenberg P, Simoes EAF, Singleton R, Sistla S, Sturm-Ramirez K, Suntronwong N, Sutanto A, Tapia MD, Thamthitiwat S, Thongpan I, Tillekeratne G, Tinoco YO, Treurnicht FK, Turner C, Turner P, van Doorn R, van Ranst M, Visseaux B, Waicharoen S, Wang J, Yoshida LM, Zar HJ (2019). Global patterns in monthly activity of influenza virus, respiratory syncytial virus, parainfluenza virus, and metapneumovirus: a systematic analysis. Lancet Glob Health.

[37] Morley C, Grimwood K, Maloney S, Ware RS (2018). Meteorological factors and respiratory syncytial virus seasonality in subtropical Australia. Epidemiol Infect.

[38] Bo Y, Guo C, Lin C, Zeng Y, Li HB, Zhang Y, Hossain MS, Chan JWM, Yeung DW, Kwok KO, Wong SYS, Lau AKH, Lao XQ (2021). Effectiveness of non-pharmaceutical interventions on COVID-19 transmission in 190 countries from 23 January to 13 April 2020. Int J Infect Dis.

[39] Li Y, Campbell H, Kulkarni D, Harpur A, Nundy M, Wang X, Nair H (2021). The temporal association of introducing and lifting non-pharmaceutical interventions with the time-varying reproduction number (R) of SARS-CoV-2: a modelling study across 131 countries. Lancet Infect Dis.

[40] Piovani D, Christodoulou MN, Hadjidemetriou A, Pantavou K, Zaza P, Bagos PG, Bonovas S, Nikolopoulos GK (2021). Effect of early application of social distancing interventions on COVID-19 mortality over the first pandemic wave: an analysis of longitudinal data from 37 countries. J Inf Secur.

[41] Imai C, Brooks WA, Chung Y, Goswami D, Anjali BA, Dewan A, Kim H, Hashizume M (2014). Tropical influenza and weather variability among children in an urban low-income population in Bangladesh. Glob Health Action.

[42] Colón-González FJ, Lake IR, Bentham G (2011). Climate variability and dengue fever in warm and humid Mexico. Am J Trop Med Hyg.

[43] Cowling BJ, Ali ST, Ng TWY, Tsang TK, Li JCM, Fong MW, Liao Q, Kwan MY, Lee SL, Chiu SS (2020). Impact assessment of non-pharmaceutical interventions against coronavirus disease 2019 and influenza in Hong Kong: an observational study. Lancet Public Health.

[44] Kuitunen I, Artama M, Mäkelä L, Backman K, Heiskanen-Kosma T, Renko M (2020). Effect of social distancing due to the COVID-19 pandemic on the incidence of viral respiratory tract infections in children in Finland during early 2020. Pediatr Infect Dis J.

[45] Machida M, Nakamura I, Saito R, Nakaya T, Hanibuchi T, Takamiya T, Odagiri Y, Fukushima N, Kikuchi H, Amagasa S, Kojima T, Watanabe H, Inoue S (2020). Changes in implementation of personal protective measures by ordinary Japanese citizens: a longitudinal study from the early phase to the community transmission phase of the COVID-19 outbreak. Int J Infect Dis.

[46] Machida M, Nakamura I, Saito R, Nakaya T, Hanibuchi T, Takamiya T, Odagiri Y, Fukushima N, Kikuchi H, Kojima T, Watanabe H, Inoue S (2020). Adoption of personal protective measures by ordinary citizens during the COVID-19 outbreak in Japan. Int J Infect Dis.

[47] Nomura S, Yoneoka D, Shi S, Tanoue Y, Kawashima T, Eguchi A, Matsuura K, Makiyama K, Ejima K, Taniguchi T, Sakamoto H, Kunishima H, Gilmour S, Nishiura H, Miyata H (2020). An assessment of self-reported COVID-19 related symptoms of 227,898 users of a social networking service in Japan: has the regional risk changed after the declaration of the state of emergency?. Lancet Reg Health – West Pac.

[48] Yoneoka D, Tanoue Y, Kawashima T, Nomura S, Shi S, Eguchi A, Ejima K, Taniguchi T, Sakamoto H, Kunishima H, Gilmour S, Nishiura H, Miyata H (2020). Large-scale epidemiological monitoring of the COVID-19 epidemic in Tokyo. Lancet Reg Health – West Pac.

[49] Sax H, Allegranzi B, Chraïti MN, Boyce J, Larson E, Pittet D (2009). The World Health Organization hand hygiene observation method. Am J Infect Control.

[50] Allegranzi B, Gayet-Ageron A, Damani N, Bengaly L, McLaws ML, Moro ML, Memish Z, Urroz O, Richet H, Storr J (2013). Global implementation of WHO's multimodal strategy for improvement of hand hygiene: a quasi-experimental study. Lancet Infect Dis.

[51] Rabie T, Curtis V (2006). Handwashing and risk of respiratory infections: a quantitative systematic review. Trop Med Int Health.

[52] Aiello AE, Coulborn RM, Perez V, Larson EL (2008). Effect of hand hygiene on infectious disease risk in the community setting: a meta-analysis. Am J Public Health.

[53] Feltes TF, Cabalka AK, Meissner HC, Piazza FM, Carlin DA, Top FH, Connor EM, Sondheimer HM (2003). Palivizumab prophylaxis reduces hospitalization due to respiratory syncytial virus in young children with hemodynamically significant congenital heart disease. J Pediatr.

[54] Dapat C, Kumaki S, Sakurai H, Nishimura H, Labayo HKM, Okamoto M, Saito M, Oshitani H (2021). Gene signature of children with severe respiratory syncytial virus infection. Pediatr Res.

[55] Schmidt WP, Aunger R, Coombes Y, Maina PM, Matiko CN, Biran A, Curtis V (2009). Determinants of handwashing practices in Kenya: the role of media exposure, poverty and infrastructure. Trop Med Int Health.

[56] Isunju JB, Schwartz K, Schouten MA, Johnson WP, van Dijk MP (2011). Socio-economic aspects of improved sanitation in slums: a review. Public Health.

[57] Miyama T, Iritani N, Nishio T, Ukai T, Satsuki Y, Miyata H, et al. Seasonal shift in epidemics of respiratory syncytial virus infection in Japan. Epidemiol Infect. 2021;149:e55. 10.1017/S0950268821000340. 10.1017/S0950268821000340 PMC8060823 33568242

[58] Wagatsuma K, Koolhof IS, Shobugawa Y, Saito R (2021). Shifts in the epidemic season of human respiratory syncytial virus associated with inbound overseas travelers and meteorological conditions in Japan, 2014-2017: an ecological study. PLoS One.

[59] Chinazzi M, Davis JT, Ajelli M, Gioannini C, Litvinova M, Merler S, Pastore YPA, Mu K, Rossi L, Sun K (2020). The effect of travel restrictions on the spread of the 2019 Novel coronavirus (COVID-19) outbreak. Science (New York, NY).

[60] Wells CR, Sah P, Moghadas SM, Pandey A, Shoukat A, Wang Y, Wang Z, Meyers LA, Singer BH, Galvani AP (2020). Impact of international travel and border control measures on the global spread of the novel 2019 coronavirus outbreak. Proc Natl Acad Sci U S A.

[61] Anzai A, Kobayashi T, Linton NM, Kinoshita R, Hayashi K, Suzuki A, et al. Assessing the impact of reduced travel on exportation dynamics of Novel Coronavirus Infection (COVID-19). J Clin Med. 2020;9(2):601. 10.3390/jcm9020601. 10.3390/jcm9020601 PMC7073579 32102279

[62] Wagatsuma K, Phyu WW, Osada H, Tang JW, Saito R. Geographic correlation between the number of COVID-19 cases and the number of overseas travelers in Japan. Jpn J Infect Dis. 2021;74(2):157-60. 10.7883/yoken.JJID.2020.471. 10.7883/yoken.JJID.2020.471 32863355

[63] Shi S, Tanaka S, Ueno R, Gilmour S, Tanoue Y, Kawashima T, Nomura S, Eguchi A, Miyata H, Yoneoka D (2020). Travel restrictions and SARS-CoV-2 transmission: an effective distance approach to estimate impact. Bull World Health Organ.

[64] Nishiura H, Wilson N, Baker MG (2009). Quarantine for pandemic influenza control at the borders of small island nations. BMC Infect Dis.

[65] Murano Y, Ueno R, Shi S, Kawashima T, Tanoue Y, Tanaka S, Nomura S, Shoji H, Shimizu T, Nguyen H, Miyata H, Gilmour S, Yoneoka D (2021). Impact of domestic travel restrictions on transmission of COVID-19 infection using public transportation network approach. Sci Rep.

[66] Juan HC, Chao CM, Lai CC, Tang HJ (2020). Decline in invasive pneumococcal disease during COVID-19 pandemic in Taiwan. J Inf Secur.

[67] Lim RH, Chow A, Ho HJ (2020). Decline in pneumococcal disease incidence in the time of COVID-19 in Singapore. J Inf Secur.

[68] Lai CC, Yu WL (2020). The COVID-19 pandemic and tuberculosis in Taiwan. J Inf Secur.

[69] Komiya K, Yamasue M, Takahashi O, Hiramatsu K, Kadota JI, Kato S (2020). The COVID-19 pandemic and the true incidence of tuberculosis in Japan. J Inf Secur.

[70] Hsu YL, Lin HC, Wei HM, Lai HC, Hwang KP (2020). One benefit of COVID-19 measures in Taiwan: the reduction of influenza infections and severe complications. Influenza Other Respir Viruses.

[71] Rana MS, Usman M, Alam MM, Mere MO, Ikram A, Zaidi SSZ, et al. Impact of COVID-19 pandemic on measles surveillance in Pakistan. J Inf Secur. 2021;82(3):414-51. 10.1016/j.jinf.2020.10.008. 10.1016/j.jinf.2020.10.008 PMC7543746 33039503

[72] Eigner U, Verstraeten T, Weil J. Decrease in norovirus infections in Germany following COVID-19 containment measures. J Inf Secur. 2021;82(6):276-316. 10.1016/j.jinf.2021.02.012. 10.1016/j.jinf.2021.02.012 PMC8711047 33581238

[73] Itaya T, Furuse Y, Jindai K (2020). Does COVID-19 infection impact on the trend of seasonal influenza infection? 11 countries and regions, from 2014 to 2020. Int J Infect Dis.

[74] Lai C-C, Chen S-Y, Yen M-Y, Lee P-I, Ko W-C, Hsueh P-R. The impact of the coronavirus disease 2019 epidemic on notifiable infectious diseases in Taiwan: a database analysis. Travel Med Infect Dis. 2021;40:101997. 10.1016/j.tmaid.2021.101997. 10.1016/j.tmaid.2021.101997 PMC7905388 33640476

[75] Hellewell J, Abbott S, Gimma A, Bosse NI, Jarvis CI, Russell TW, Munday JD, Kucharski AJ, Edmunds WJ, Funk S (2020). Feasibility of controlling COVID-19 outbreaks by isolation of cases and contacts. Lancet Glob Health.

[76] Ferdous MZ, Islam MS, Sikder MT, Mosaddek ASM, Zegarra-Valdivia JA, Gozal D (2020). Knowledge, attitude, and practice regarding COVID-19 outbreak in Bangladesh: an online-based cross-sectional study. PLoS One.

[77] Cheng VC, Wong SC, Chuang VW, So SY, Chen JH, Sridhar S, Chan JF, Hung IF, Ho PL, To KK (2020). The role of community-wide wearing of face mask for control of coronavirus disease 2019 (COVID-19) epidemic due to SARS-CoV-2. J Inf Secur.

[78] Prem K, Liu Y, Russell TW, Kucharski AJ, Eggo RM, Davies N, Jit M, Klepac P (2020). The effect of control strategies to reduce social mixing on outcomes of the COVID-19 epidemic in Wuhan, China: a modelling study. Lancet Public Health.

[79] Heikkinen T, Ojala E, Waris M (2017). Clinical and socioeconomic burden of respiratory syncytial virus infection in children. J Infect Dis.

[80] Duvvuri VR, Granados A, Rosenfeld P, Bahl J, Eshaghi A, Gubbay JB (2015). Genetic diversity and evolutionary insights of respiratory syncytial virus a ON1 genotype: global and local transmission dynamics. Sci Rep.

[81] Eshaghi A, Duvvuri VR, Lai R, Nadarajah JT, Li A, Patel SN, Low DE, Gubbay JB (2012). Genetic variability of human respiratory syncytial virus a strains circulating in Ontario: a novel genotype with a 72 nucleotide G gene duplication. PLoS One.

[82] Abou-El-Hassan H, Massaad E, Soudani N, Assaf-Casals A, Shaker R, Lteif Khoury M, Ghanem S, Karam M, Andary R, Saito R (2019). Detection of ON1 and novel genotypes of human respiratory syncytial virus and emergence of palivizumab resistance in Lebanon. PLoS One.

[83] Yusuf S, Piedimonte G, Auais A, Demmler G, Krishnan S, Van Caeseele P, Singleton R, Broor S, Parveen S, Avendano L (2007). The relationship of meteorological conditions to the epidemic activity of respiratory syncytial virus. Epidemiol Infect.

[84] Ali ST, Tam CC, Cowling BJ, Yeo KT, Yung CF (2020). Meteorological drivers of respiratory syncytial virus infections in Singapore. Sci Rep.

[85] Tang JW, Loh TP (2014). Correlations between climate factors and incidence--a contributor to RSV seasonality. Rev Med Virol.

[86] Furuse Y, Sando E, Tsuchiya N, Miyahara R, Yasuda I, Ko YK, Saito M, Morimoto K, Imamura T, Shobugawa Y, Nagata S, Jindai K, Imamura T, Sunagawa T, Suzuki M, Nishiura H, Oshitani H (2020). Clusters of coronavirus disease in communities, Japan, January-April 2020. Emerg Infect Dis.

[87] Miyahara R, Tsuchiya N, Yasuda I, Ko Y, Furuse Y, Sando E, et al. Familial clusters of Coronavirus Disease in 10 prefectures, Japan, February–May 2020. Emerg Infect Dis J. 2021;27(3):915-8. 10.3201/eid2703.203882. 10.3201/eid2703.203882 PMC7920650 33622475

[88] Furuse Y, Ko YK, Saito M, Shobugawa Y, Jindai K, Saito T, Nishiura H, Sunagawa T, Suzuki M, Oshitani H (2020). Epidemiology of COVID-19 outbreak in Japan, from January-March 2020. Jpn J Infect Dis.

[89] Feng L, Zhang T, Wang Q, Xie Y, Peng Z, Zheng J, Qin Y, Zhang M, Lai S, Wang D, Feng Z, Li Z, Gao GF (2021). Impact of COVID-19 outbreaks and interventions on influenza in China and the United States. Nat Commun.

[90] Tan HMJ, Tan MS, Chang ZY, Tan KT, Ee GLA, Ng CCD, Hwang YKW, Koh YLE, Low YPS, Tan NC (2021). The impact of COVID-19 pandemic on the health-seeking behaviour of an Asian population with acute respiratory infections in a densely populated community. BMC Public Health.

[91] Rizvi RF, Craig KJT, Hekmat R, Reyes F, South B, Rosario B, Kassler WJ, Jackson GP (2021). Effectiveness of non-pharmaceutical interventions related to social distancing on respiratory viral infectious disease outcomes: a rapid evidence-based review and meta-analysis. SAGE Open Med.

