# Supplementary material for: Decreased human respiratory syncytial virus activity during the COVID-19 pandemic in Japan: an ecological time-series analysis
Source: BMC Infect Dis. 2021 Aug 3;21:734. doi: 10.1186/s12879-021-06461-5 (PMC8329631; doi:10.1186/s12879-021-06461-5)
Supplement: Supplementary file 1 — Additional file 1 : Figure S1. Probability distribution of monthly HRSV activity at national level in Japan during 2014–2020. Table S1. Spearman’s rank-order correlation coefficients matrix of monthly variables included in this study during 2014–2020. Table S2. Spearman’s rank-order correlation coefficients showing the lag effect for individual NPI indicators and meteorological conditions. Table S3. Regression coefficient for HRSV activity: the results of a multivariate analysis. Table S4. Regression coefficient for HRSV activity: the results of a multivariate analysis. Table S5. Regression coefficient for HRSV activity: the results of a multivariate analysis. [file 12879_2021_6461_MOESM1_ESM.docx]

## **Supplementary information**

**Additional file 1 for ‘Decreased human respiratory syncytial virus activity during the COVID-19 pandemic in Japan: an ecological time-series analysis’**

**Authors:**

Keita Wagatsuma^1^, Iain S. Koolhof^2^, Yugo Shobugawa^3^, and Reiko Saito^1^

**Affiliations:**

^1^ Division of International Health (Public Health), Graduate School of Medical and Dental Sciences, Niigata University, Niigata, Japan

^2^ College of Health and Medicine, School of Medicine, University of Tasmania, Hobart, Australia

^3^ Department of Active Ageing (donated by Tokamachi city, Niigata, Japan), Graduate School of Medical and Dental Sciences, Niigata University, Niigata, Japan

**E-mail addresses:**

Keita Wagatsuma ([waga@med.niigata-u.ac.jp](mailto:waga@med.niigata-u.ac.jp))

Iain S. Koolhof ([koolhofi@utas.edu.au](mailto:koolhofi@utas.edu.au))

Yugo Shobugawa ([yugo@med.niigata-u.ac.jp](mailto:yugo@med.niigata-u.ac.jp))

Reiko Saito ([jasmine@med.niigata-u.ac.jp](mailto:jasmine@med.niigata-u.ac.jp))

**Corresponding author:**

Keita Wagatsuma

Division of International Health (Public Health)

Graduate School of Medical and Dental Sciences, Niigata University

1-757 Asahimachi dori, Chuo-ku, Niigata City, Niigata 951-8510, Japan

Tel: +81-25-227-2129; Fax +81-25-227-0765

E-mail address: [waga@med.niigata-u.ac.jp](mailto:waga@med.niigata-u.ac.jp)


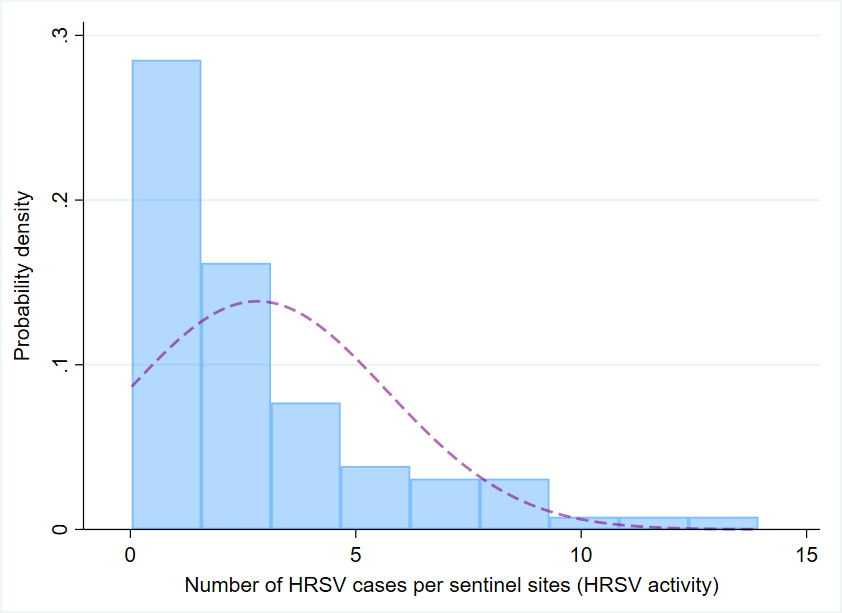


**Figure S1. Probability distribution of monthly HRSV activity at national level in Japan during 2014–2020.** The average monthly number of HRSV cases per sentinel site at the national level in Japan was 2.8 (standard deviation [SD] 2.8). These data did not follow a normal distribution (Shapiro-Wilk test, *p*<0.001).

**Table S1. Spearman**'**s rank-order correlation coefficients matrix of monthly variables included in this study during 2014–2020.**

| Variable | Unit | 1 | 2 | 3 | 4 | 5 | 6 |
| --- | --- | --- | --- | --- | --- | --- | --- |
| 1. Retail sales of hand hygiene products^a^ | yen | 1.00 |  |  |  |  |  |
| 2. Domestic airline passenger arrivals^b^ | person | –0.18 | 1.00 |  |  |  |  |
| 3. International airline passengers arrivals^c^ | person | –0.13 | 0.70^***^ | 1.00 |  |  |  |
| 4. Average temperature | ℃ | 0.07 | 0.35^***^ | 0.07 | 1.00 |  |  |
| 5. Relative humidity | % | 0.15 | 0.25^*^ | 0.03 | 0.66^*^ | 1.00 |  |
| 6. Year | year | 0.53^***^ | –0.05 | 0.26^*^ | 0.03 | 0.07 | 1.00 |

^a^ Retail sales of hand hygiene products per ¥1 billion (unit: yen).

^b^ Number of domestic airline passenger arrivals per 1,000 population (unit: person).

^c^ Number of international airline passenger arrivals per 1,000 population (unit: person).

^*^ *p*<0.05.

^**^ *p*<0.01.

^***^ *p*<0.001.

**Table S2. Spearman**'**s rank-order correlation coefficients showing the lag effect for individual NPI indicators and meteorological conditions.**

| Variable | Unit | Lag 0 (month) | Lag 1 (month) | Lag 2 (month) | Lag 3 (month) | Lag 4 (month) |
| --- | --- | --- | --- | --- | --- | --- |
| Retail sales of hand hygiene products^a^ | yen | –0.26^*^ | –0.22^*^ | –0.17 | –0.20 | –0.13 |
| Domestic airline passengers arrivals^b^ | person | 0.60^***^ | 0.72^***^ | 0.68^***^ | 0.52^***^ | 0.39^***^ |
| International airline passengers arrivals^c^ | person | 0.43^***^ | 0.47^***^ | 0.47^***^ | 0.41^***^ | 0.36^***^ |
| Average temperature | ℃ | –0.03 | 0.26^*^ | 0.47^***^ | 0.55^***^ | 0.50^***^ |
| Relative humidity | % | 0.20 | 0.42^f^ | 0.47^***^ | 0.36^***^ | 0.13 |

^a^ Retail sales of hand hygiene products per ¥1 billion (unit: yen).

^b^ Number of domestic airline passenger arrivals per 1,000 population (unit: person).

^c^ Number of international airline passenger arrivals per 1,000 population (unit: person).

^*^ *p*<0.05.

^**^ *p*<0.01.

^***^ *p*<0.001.

**Table S3. Regression coefficient for HRSV activity: the results of a multivariate analysis.**

|  |  | Model 1^b^ | |  | Model 2^c^ | |  | Model 3^d^ | |
| --- | --- | --- | --- | --- | --- | --- | --- | --- | --- |
| Variable | Lag (months)^a^ | Coef. | *p* |  | Coef. | *p* |  | Coef. | *p* |
| NPI indicator | | | | | | | | | |
| Retail sales of hand hygiene products^e^ | 0–2 | –0.44 | <0.001 |  |  |  |  |  |  |
|  | 0–3 |  |  |  | –0.55 | <0.001 |  |  |  |
|  | 0–4 |  |  |  |  |  |  | –0.58 | <0.001 |

Abbreviations: Coef., regression coefficient.

^a^ Moving average.

^b^ Generalized linear gamma regression model adjusted for the number of monthly retail sales of hand hygiene products per ¥1 billion at lag of 0–2 months, monthly average temperature at lag of 3–4 months, monthly relative humidity at lag of 1–2 months, and year variables (years 2014, 2015, 2016, 2017, 2018, 2019, and 2020).

^c^ Generalized linear gamma regression model adjusted for the monthly retail sales of hand hygiene products per ¥1 billion at lag of 0–3 months, monthly average temperature at lag of 3–4 months, monthly relative humidity at lag of 1–2 months, and year variables (years 2014, 2015, 2016, 2017, 2018, 2019, and 2020).

^d^ Generalized linear gamma regression model adjusted for the number of monthly retail sales of hand hygiene products per ¥1 billion at lag of 0–4 months, monthly average temperature at lag of 3–4 months, monthly relative humidity at lag of 1–2 months, and year variables (years 2014, 2015, 2016, 2017, 2018, 2019, and 2020).

^e^ Retail sales of hand hygiene products per ¥1 billion (unit: yen).

**Table S4. Regression coefficient for HRSV activity: the results of a multivariate analysis.**

|  |  | Model 1^b^ | |  | Model 2^c^ | |
| --- | --- | --- | --- | --- | --- | --- |
| Variable | Lag (months)^a^ | Coef. | *p* |  | Coef. | *p* |
| NPI indicator | | | | | | |
| Domestic airline passenger arrivals^d^ | 1–3 | 3.6×10^−4^ | <0.001 |  |  |  |
|  | 1–4 |  |  |  | 3.5×10^−4^ | <0.001 |

Abbreviations: Coef., regression coefficient.

^a^ Moving average.

^b^ Generalized linear gamma regression model adjusted for monthly number of domestic airline passenger arrivals per 1,000 population at lag of 1–3 months, monthly average temperature at lag of 3–4 months, monthly relative humidity at lag of 1–2 months, and year variables (years 2014, 2015, 2016, 2017, 2018, 2019, and 2020).

^c^ Generalized linear gamma regression model adjusted for monthly number of domestic airline passenger arrivals per 1,000 population at lag of 1–4 months, monthly average temperature at lag of 3–4 months, monthly relative humidity at lag of 1–2 months, and year variables (years 2014, 2015, 2016, 2017, 2018, 2019, and 2020).

^d^ Number of domestic airline passenger arrivals per 1,000 population (unit: person).

**Table S5. Regression coefficient for HRSV activity: the results of a multivariate analysis.**

|  |  | Model 1^b^ | |  | Model 2^c^ | |
| --- | --- | --- | --- | --- | --- | --- |
| Variable | Lag (months)^a^ | Coef. | *p* |  | Coef. | *p* |
| International airline passenger arrivals^d^ | 1–3 | 1.1×10^−3^ | <0.001 |  |  |  |
|  | 1–4 |  |  |  | 1.1×10^−3^ | <0.001 |

Abbreviations: Coef., regression coefficient.

^a^ Moving average.

^b^ Generalized linear gamma regression model adjusted for number of international airline passenger arrivals per 1,000 population at lag of 1–3 months, monthly average temperature at lag of 3–4 months, monthly relative humidity at lag of 1–2 months, and year variables (years 2014, 2015, 2016, 2017, 2018, 2019, and 2020).

^c^ Generalized linear gamma regression model adjusted for number of international airline passenger arrivals per 1,000 population at lag of 1–4 months, monthly average temperature at lag of 3–4 months, monthly relative humidity at lag of 1–2 months, and year variables (years 2014, 2015, 2016, 2017, 2018, 2019, and 2020).

^d^ Number of international airline passenger arrivals per 1,000 population (unit: person).
